# Roles of Gasotransmitters in Synaptic Plasticity and Neuropsychiatric Conditions

**DOI:** 10.1155/2018/1824713

**Published:** 2018-05-06

**Authors:** Ulfuara Shefa, Dokyoung Kim, Min-Sik Kim, Na Young Jeong, Junyang Jung

**Affiliations:** ^1^Department of Biomedical Science, Graduate School, Kyung Hee University, 26 Kyungheedae-ro, Dongdaemun-gu, Seoul 02447, Republic of Korea; ^2^Department of Anatomy and Neurobiology, College of Medicine, Kyung Hee University, 26 Kyungheedae-ro, Dongdaemun-gu, Seoul 02447, Republic of Korea; ^3^Department of Applied Chemistry, College of Applied Science, Kyung Hee University, Deogyeong-daero, Giheung-gu, Yongin-si, Gyeonggi-do 17104, Republic of Korea; ^4^Department of Anatomy and Cell Biology, College of Medicine, Dong-A University, 32 Daesingongwon-ro, Seo-gu, Busan 49201, Republic of Korea; ^5^East-West Medical Research Institute, Kyung Hee University, 26 Kyungheedae-ro, Dongdaemun-gu, 13 Seoul 02447, Republic of Korea

## Abstract

Synaptic plasticity is important for maintaining normal neuronal activity and proper neuronal functioning in the nervous system. It is crucial for regulating synaptic transmission or electrical signal transduction to neuronal networks, for sharing essential information among neurons, and for maintaining homeostasis in the body. Moreover, changes in synaptic or neural plasticity are associated with many neuropsychiatric conditions, such as schizophrenia (SCZ), bipolar disorder (BP), major depressive disorder (MDD), and Alzheimer's disease (AD). The improper maintenance of neural plasticity causes incorrect neurotransmitter transmission, which can also cause neuropsychiatric conditions. Gas neurotransmitters (gasotransmitters), such as hydrogen sulfide (H_2_S), nitric oxide (NO), and carbon monoxide (CO), play roles in maintaining synaptic plasticity and in helping to restore such plasticity in the neuronal architecture in the central nervous system (CNS). Indeed, the upregulation or downregulation of these gasotransmitters may cause neuropsychiatric conditions, and their amelioration may restore synaptic plasticity and proper neuronal functioning and thereby improve such conditions. Understanding the specific molecular mechanisms underpinning these effects can help identify ways to treat these neuropsychiatric conditions.

## 1. Introduction

The polish psychologist Konorski (1948) first used the term “synaptic plasticity” to describe consistent and activity-dependent changes in synaptic strength [1]. Synaptic plasticity is an experience-dependent change in synaptic strength [2]. Changes in synaptic strength are essential for information storage during memory formation [3], and recent work has revealed that synaptic plasticity also plays roles in other adaptive responses, including mood stability, drug addiction, and chronic pain [4]. The mechanisms underpinning synaptic plasticity are broadly linked to long-term memory. Synapse modifications are commonly monitored by two important phenomena: long-term potentiation (LTP) and long-term depression (LTD), which cause an increase or a reduction in synaptic strength, respectively. LTP and LTD also have roles in memory and learning [1]. Neurotransmitters are the chemical messengers that activate, amplify, and harmonize signals between neurons and other cells in the body. Neuronal functions rely on a balance between the number of relevant excitatory and inhibitory processes, which may happen individually or concomitantly [5].

The gas neurotransmitters (gasotransmitters) in our body include hydrogen sulfide (H_2_S), nitric oxide (NO), and carbon monoxide (CO); they play essential roles in normal physiology and under pathological conditions. H_2_S is a member of the gasotransmitter family that is associated with the maintenance of neuronal plasticity, excitability, and the central nervous system (CNS) [6]. *N*-Methyl-D-aspartate (NMDA) receptors are targets of H_2_S in the brain; H_2_S potentiates the activity of NMDA receptors and facilitates the induction of hippocampal LTP [7]. Hence, a recent study demonstrated that H_2_S could reduce NMDA receptor-mediated currents in pyramidal neurons of the Cornu Ammonis (CA3) region of neonatal hippocampal slices [6]. NO is a ubiquitous signaling molecule in the brain as well as in other organs in the body, and many reviews have described its role in retrograde signaling [8], cellular function, synaptic plasticity [9], development, excitotoxicity, blood flow, and mental health [10]. NO inhibits the activity of NMDA receptors and thereby reduces the effects of glutamate and induces changes in neural transmission. A reduction in NMDA receptor (NMDAR) expression is associated with the change in synaptic plasticity driven by the age-related conditions in sensory input, demonstrating age-related impairment in the function of the NMDAR/NO signaling pathway in the CNS [11]. Physiologically, CO is generated by two heme oxygenases, hemeoxygenase-1 (HO-1) and hemeoxygenase-2 (HO-2), which catalyze the catabolism of heme groups [12]. HO-2 is concentrated in hippocampal pyramidal cells; therefore, CO might be a candidate retrograde messenger for LTP as the HO inhibitor zinc protoporphyrin IX (Znpp-9) blocks the induction of LTP in hippocampal slices [13].

In this review, we will briefly describe the role of synaptic plasticity in normal neuronal functioning or homeostasis and examine how alterations in neural plasticity hamper the release and signaling of neurotransmitters, such as H_2_S, NO, and CO, to cause neuropsychiatric conditions, such as major depressive disorder (MDD), schizophrenia (SCZ), bipolar disorder (BD), and Alzheimer's disease (AD). We will also address how the upregulation or downregulation of these gasotransmitters affects disease progression. Finally, we will discuss therapeutic options and how, by understanding the pathways through which alterations in neural plasticity cause disorders, we can target the responsible molecules to prevent these neuropsychiatric conditions.

## 2. Synaptic Plasticity and Its Neurobiology

Synaptic plasticity in the mature nervous system includes structural and morphological modifications, such as dendritic spine growth and synaptogenesis [14]. These modifications are the cellular response to the changes in neuronal activity that are thought to be responsible for learning and memory [15]. The mitochondria present in axonal terminals and the dendrites of neurons play important roles in synaptic activity [16].

Various neurotransmitter receptors are functionally linked with protein kinases as well as other G-proteins that modulate cascades of molecules which in turn maintain essential cellular functions [17]. As an example, the mitogen-activated protein kinase- (MAPK-) related pathway activates transcription factors associated with learning, memory, and cell proliferation as well as apoptosis. This pathway intricates extracellular stimuli via the phosphorylation of c-Jun *N*-terminal kinase (JNK), extracellular signal-regulated kinase (ERK), and P38 as well as other kinases. Similarly, the MAPK-involved pathway and 3′-5′-cyclic adenosine monophosphate- (cAMP-) associated pathway are also jointed to activate neurotransmitter receptors as well as modulate cellular functions via the activation of protein kinase A (PKA), exchange protein stimulated by cAMP (EPAC), and other molecules [18]. Modifications of the MAPK-and c-AMP-related signaling pathways may affect intracellular Ca^2+^ levels, neurotransmitter receptors, transcription factors, and the cross link between signaling pathways as well as other biological functions which is essential for neuroplasticity [19].

**Figure 1:**
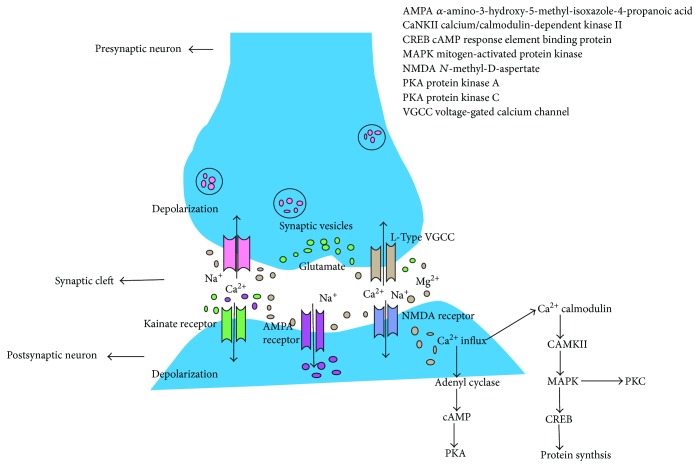
Transmission of signals through the synaptic junctions. Signals or impulses at the presynaptic terminal trigger the release of glutamate that binds to glutamate receptors at the postsynaptic membrane. Activation of *α*-amino-3-hydroxy-5-methyl-isoxazole-4-propanoic acid (AMPA) as well as kainate receptors which subsequently transport sodium ions that trigger postsynaptic depolarization. As membrane potential changes, it initiates the release of magnesium ions which blocks *N*-methyl-D-aspartate (NMDA) receptors. Influx of calcium via NMDA channels sets off a chain of events which establish long-term potentiation. Kainate receptors at the presynaptic end also seem to facilitate synaptic transmission at particular synapses by accumulating neurotransmitter release.

Structurally, synaptic plasticity involves the insertion into or the removal of *α*-amino-3-hydroxy-5-methyl-isoxazole-4-propanoic acid (AMPA) receptors from the postsynaptic membrane and the enlargement or shrinkage of the dendritic spines where most excitatory synapses (~90%) are located [20]. Functionally, synaptic plasticity is regarded as the LTP or LTD of synaptic strength, demonstrating changes in conductance via AMPA receptors (AMPARs) in the postsynaptic membrane. During the period of plasticity, NMDAR activation allows calcium ions (Ca^2+^) to cross the postsynaptic membrane and initiate intracellular signaling cascades (Figure 1). These cascades trigger gene transcription, AMPAR trafficking via action dynamics, reorganization of the cytoskeleton, and enlargement or elimination of dendritic spines. The integrity of the synaptic structure, AMPAR trafficking, and dendritic spine dynamics are all pivotal for generating lasting synaptic plasticity changes (Figure 1) [20].

On the neurobiological level, learning and memory depend on regulated signaling processes at synapses as well as synaptic communication between neurons and other cellular partners. Molecular-level plasticity can be driven by increased expression of plasticity-related genes, such as brain-derived neurotrophic factor (BDNF), calcium/calmodulin kinase II (CaMKII), and cyclic AMP (cAMP) response element binding (CREB) protein, as well as by enhanced surface expression of glutamatergic AMPARs and NMDARs. The neurotrophin BDNF and its signaling partners are the main regulators of synaptic plasticity, a biological process that regulates synaptic strength via neuronal activity [21]. Different neuromodulatory factors affect neuronal plasticity such as BDNF which may serve as a real mediator rather than simply a modulator of synaptic plasticity and synaptic communication [21]. Moreover, BDNF and neurotransmitter signaling cascades can work together in close temporal association to induce immediate and guided effects on synaptic plasticity [22]. However, more attention has been given to BDNF because specifically interfering with BDNF-related signaling is a key strategy for initiating neuronal and functionally restorative treatments for neurological and psychiatric disorders [23].

AMPARs and NMDARs are the receptors that synergize at postsynaptic terminals to facilitate different forms of synaptic plasticity (Figure 1). Constant activation of AMPARs by a series of impulses arriving at presynaptic terminals leads to depolarization of the presynaptic membrane, which removes the magnesium ions (Mg^2+^) that are obstructed at NMDARs [24]. Hence, consistent with the Hebb hypothesis, the simultaneous excitation of pre- and postsynaptic neurons expedites the gating of NMDA channels and strengthens the synapse. This is a crucial feature of NMDA channels that is specifically associated with synaptic plasticity and its high permeability to calcium. Consequently, the second messenger calcium modulates a battery of signaling pathways and the responses that collectively elicit synaptic modification [25]. NMDARs are also involved in synaptic plasticity, but the situation is far more complex, as many forms of LTP are imparted by diverse inputs in various neurons. One intriguing case is that of activity-dependent synaptic plasticity, which is stimulated by presynaptic NMDA channels. In the lateral nucleus of the amygdala, neuronal activity induces a form of LTP that requires NMDARs but is independent of postsynaptic activity [26]. These observations suggest that NMDA functions, which are critical for learning and memory, are not limited to postsynaptic terminals [25].

Voltage-gated calcium channels (VGCCs) play roles in signal transduction between neurons as well as in various forms of synaptic plasticity. Interestingly, patients with AD express higher levels of L-type VGCC in the hippocampus compared with control subjects [27]. Activity-dependent neuroplastic mechanisms in the hippocampus that are fundamental to learning and memory, such as LTP, can be altered by the preceding synaptic activity. The concept of neuroplasticity has been implicated in various neurological and psychiatric diseases and conditions, including AD, depression, SCZ, aging, epilepsy, neurodevelopmental disorders, metabolic disorders, and neuroinflammation, such as multiple sclerosis (MS) [28].

In summary, various receptors and ion channels regulate synaptic plasticity, proper neural functioning, and neural homeostasis.

## 3. Gasotransmitters (H_2_S, NO, and CO) in the Nervous System

Three main gasotransmitters that play crucial physiological roles in the body were discovered recently: H_2_S, CO, and NO. These gasotransmitters also have pathologic functions [29].

### 3.1. H_2_S

H_2_S is an essential signaling molecule with many homeostatic functions, such as neurotransmission and neuromodulation; it is also associated with learning, memory, and nociception [29]. *In vivo*, five enzymes are associated with H_2_S synthesis: cystathionine *β*-synthase (CBS), 3-mercaptopyruvate sulfurtransferase (3-MST), cystathionine *γ*-lyase (CSE), cysteine aminotransferase (CAT), and D-amino acid oxidase (DAAO) [30]. CBS is thought to be the major H_2_S-producing enzyme in the brain [31].

Novel signaling molecules linked to polysulfide (H_2_S_n_) such as hydrogen persulfide and trisulfide (H_2_S_2_ and H_2_S_3_) help maintain neuronal transmission, vascular tone, cytoprotection, inflammation, and oxygen-sensing. A recent study reported that H_2_S_2_, H_2_S_3_, and H_2_S are generated by 3-MST [32]. H_2_S_2_ and H_2_S_3_ are also produced via an interaction between H_2_S and NO. H_2_S_n_ performs additional physiological functions, such as stimulating transient receptor potential ankyrin 1 (TRPA1) channels to impart Ca^2+^ influx in astrocytes [33, 34] and dorsal root ganglion (DRG) neurons [35]. Additionally, H_2_S_2_ along with H_2_S_3_ shields neuronal cells from oxidative as well as carbonyl stresses through exerting reduced synthesis of glutathione, which is dependent on the nuclear factor erythroid 2-related factor 2 (Nrf2) [36]. Hylin and Wood demonstrated that cysteine residues in proteins can be persulfurated in the presence of 3-mercaptopyruvate (3-MP), a substrate of 3-MST [37]. Another potential mechanism of persulfuration involves H_2_S_2_ and H_2_S_3_ generated by 3-MST, which promptly interact with free cysteine and glutathione (GSH) to generate cysteine-persulfide (Cys-SSH) and glutathione-persulfide (GSSH) and also react with the cysteine residues in proteins to produce persulfurated proteins. Alternatively, 3-MST can transfer sulfur from 3-MP to cysteine, GSH, H_2_S, and cysteine residues to generate Cys-SSH, GSSH, H_2_S_2_, and persulfurated proteins. It is possible that these pathways proceed together to generate persulfurated species [38].

H_2_S accelerates the initiation of hippocampal LTP, which is a synaptic model of memory development, by increasing the function of NMDARs [7]. CBS is expressed in the brain, and the neuronal activity of H_2_S stimulates the flow of Ca^2+^ between astrocytes and neurons to adjust synaptic function [39]. The responses of astrocytes to H_2_S are suppressed by wide-spectrum transient receptor potential (TRP) channel blockers, lanthanide ions (La^3+^), gadolinium ion (Gd^3+^), and ruthenium red (RR). In a 2013 study, Kimura et al. showed that polysulfide induces Ca^2+^ inflow by stimulating transitory receptor potential TRPA1 channels in rat astrocytes [33]. The optimum activity was imparted at 0.5 *μ*M, which is 1/130 of the concentration of H_2_S needed to obtain feedback of similar magnitude. Additionally, TRPA1-specific agonists, allyl isothiocyanate, and cinnamaldehyde induced Ca^2+^ inflow, whereas responses to polysulfides were suppressed by the TRAP1-specific inhibitors HC-030031 and AP-18 as well as by TRAP1-specific small interfering RNA (siRNA). This study demonstrated that polysulfides are required for the H_2_S-derived signaling molecules that activate TRP channels in the brain [33].

Kimura demonstrated that exogenous H_2_S expedites the induction of hippocampal LTP by increasing NMDAR activity [40]. For example, an H_2_S donor enhanced NMDAR-mediated currents in the entorhinal cortex and the potentiating effect of exogenous H_2_S on NMDAR-dependent LTP has been revealed that physiological role of endogenous sulfhydration in plasticity [41]. Another recent study identified a crucial role for activity-dependent sulfhydration in D-serine-dependent synaptic plasticity. Specifically, neuronal activity facilitated H_2_S production and sulfhydrated serine racemase (SR) formation, and use of an H_2_S donor enhanced hippocampal D-serine availability, expedited hippocampal LTP via a D-serine-dependent pathway, and slowed age-related LTP impairment [41]. In this study, H_2_S levels and SR sulfhydration were reduced significantly in aged rats. As H_2_S is an important reducing agent, these changes restored D-serine levels in the hippocampus of aged rats and replenished the deficits in D-serine-dependent plasticity. Additionally, endogenous H_2_S signals protected against the age-associated impairment of synaptic plasticity [41]. The results of this study suggest that H_2_S-linked sulfhydration plays an essential role in D-serine-dependent synaptic plasticity, probably by regulating SR activation. Therefore, therapies that involve inhaled H_2_S or compounds that moderately raise brain H_2_S levels may be effective for the treatment of age-associated memory impairment [41].

### 3.2. NO

NO is regarded as a chemical transmitter which has essential functions in the mammalian central as well as peripheral nervous system [42]. NO is a gaseous molecule that can passively cross cell membranes via diffusion. It is generated by the conversion of the amino acid L-arginine into L-citrulline via the enzyme NO synthase (NOS) and inducible NOS (iNOS). Constitutive nitric oxide synthase (cNOS), or type I, is the neuronal NOS (nNOS), and it is expressed at high levels in the brain, especially in the cortex [43]. Additionally, type III or endothelial NOS (eNOS) is expressed in the endothelium. cNOS generates low levels of NO (nM range) for a short duration (seconds–minutes) under regular physiological conditions because it needs to bind to calmodulin, which occurs only while local calcium levels are increased [43].

NO is the second mediator that can activate NMDARs, which are a subtype of glutamatergic receptors [44]. NMDARs are related to the NO system. NMDAR activation persistently enhances the activity of neuronal nitric oxide synthase (nNOS) in the neuronal cytoplasm. It then catalyzes the generation of endogenous NO from L-arginine followed by the enhanced release of NO from neurons [45, 46]. Activation of these receptors by glutamate stimulates the calcium influx into cells and the generation of NO by NOS, which rapidly stimulates guanylate cyclase and increases cyclic guanosine monophosphate (cGMP) synthesis [47]. The concentration of NO reflects glutamatergic neurotransmission [48]. Hence, other glutamate receptors, such as AMPA, can also produce NO; this pathway modulates the release of glutamate and dopamine. AMPARs are important ion channels that have four subunits that operate like NMDARs, such as Glu1–4 or GluA-D [49]. Nevertheless, AMPAR trafficking, expression, and S-nitrosylation activity are maintained by NO. An ATPase named *N*-ethylmaleimide-sensitive factor (NSF) is enriched in neurons which binds with GluR2, stabilizing or recycling AMPARs with postsynaptic membranes [50]. Physiologically, synaptic NSF is S-nitrosylated by endogenous, neuronally derived NO in the mouse brain. Activation of NMDAR increases the binding of NSF to GluR2, as well as the surface insertion of GluR2. Together, these studies revealed a NO-sensitive pathway involving NMDARs and AMPARs. In particular, NMDARs stimulate NO generation, which enhances NSF S-nitrosylation, stimulates its association with GluR2, and increases the surface expression of GluR2-containing AMPARs. However, the direct S-nitrosylation activity of AMPARs has not been studied [50]. Additionally, NO is associated with the storage, uptake, and release of mediators, such as acetylcholine, noradrenaline, gamma amino butyric acid (GABA), taurine, and a glycine [51]. NO can stimulate its own extrasynaptic receptors, which are located some distance from sites of NO synthesis. In addition, NO is associated with the process of development of the nervous system [8]. For example, nNOS-containing neurons actively participate in the rostral path of neuroblast migration, which involves new synaptic connections and influences neurogenesis [52]. Astrocyte migration is also regulated by the release of NO under the actions of iNOS. NO is also recognized as critical for the formation of synapses and the growth of nerve fibers [53].

### 3.3. CO

CO is a new gaseous neuromodulatory agent that functions as a neurotransmitter or neuromodulator [54]. CO is produced during heme metabolism by HO-1 and HO-2; HO-1 is an inducible enzyme, and HO-2 is constitutively expressed. HO is the enzyme responsible for CO synthesis *in vivo*; it catalyzes the metabolism of heme to biliverdin, free iron, and CO [55]. There are three distinct HO isoforms: HO-1, HO-2, and HO-3. Of these, HO-1 and HO-2 are the most studied and best known [56] and are expressed in many tissues, including neural tissue [57]. The CO generated from heme by HO stimulates soluble guanylate cyclase activity, which promotes an increase in cGMP in neurons as well as cardiovascular functions [58]. CO may also have involved in biological activities via alternative pathways, such as the activation of cyclooxygenase, which participates in fever generation by acting on the CNS [54]. In the CNS, the CO/heme oxygenase axis plays a vital role in processes associated with cytoprotection, vasomodulation, neuroinflammation, cell death, metabolism, and cellular redox responses [59]. CO was first recognized as a neurotransmitter by Verma et al. [60]. Their research led to broad investigations into CO, heme oxygenase, and the exogenous administration of CO as a method of imparting neuroprotection and regulating tissue homeostasis in response to pathophysiological conditions, such as cerebral ischemia, cerebrovasodilation, and neurodegenerative diseases [61]. In neurons, CO-induced cGMP generation helps protect from cell death, and NO signaling is associated with the anti-inflammatory effects of CO in microglia [62].

Semiquantitative cytokine profiling of cell lysates and conditioned culture medium demonstrated increased vascular endothelial growth factor (VEGF) levels in CO-treated cultures (cell lysates) compared with controls. This is consistent with other experiments describing that CO increases VEGF levels in astrocytes and cardiomyocytes [63]. Surprisingly, a decrease in neurotrophin-3 and an increase in neurotrophin-4 levels were found in lysates from cells treated with CO. Although no studies have assessed the effects of CO treatment on neurotrophin-3 and neurotrophin-4, both neurotrophins are associated with neuronal growth and synapse formation, maturation, and plasticity. Additionally, neurotrophin-3 is expressed in neural stem cells (NSCs), and it stimulates neuronal differentiation and survival [62].

In conclusion, various gasotransmitters, such as H_2_S, NO, and CO, have potential roles in maintaining synaptic plasticity in the nervous system.

## 4. Role of Gasotransmitters in Neuropsychiatric Disease

### 4.1. MDD

MDD is a lifelong catastrophic mental disorder with high rates of morbidity and mortality [64]. The lifelong chronic–recurrent persistence of MDD is associated with very high economic and social burdens [65]. It is expected to be the second leading cause of disability worldwide by the World Health Organization (WHO) by 2020 [66]. The two gasotransmitters such as H_2_S and NO are found to have some functions in MDD.

#### 4.1.1. MDD and H_2_S

**Figure 2:**
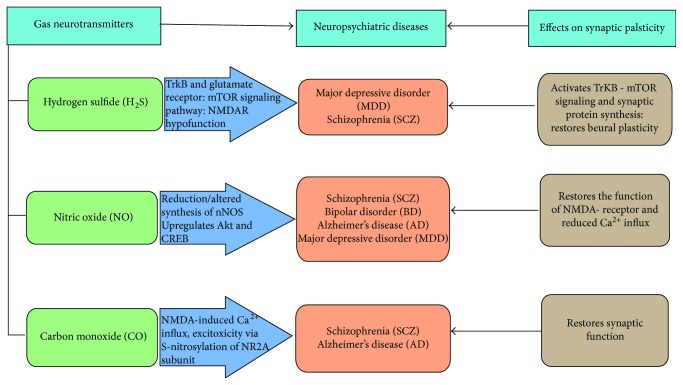
Role of gas neurotransmitters in neuropsychiatric diseases. The three gas neurotransmitters such as hydrogen sulfide (H_2_S), nitric oxide (NO), and carbon dioxide (CO) have a role in neuropsychiatric conditions such as major depressive disorder (MDD), schizophrenia (SCZ), bipolar disorder (BD), and Alzheimer's disease (AD) to maintain proper synaptic plasticity as well as neural homeostasis. H_2_S has a role in tropomyosin receptor kinase B (TrKB) and glutamate as well as mechanistic targets of rapamycin (mTOR) signaling pathways, and it activates the TrKB-mTOR signaling pathway as well as synaptic protein in MDD. NO has a role in the regulation of altered synthesis of nNOS as well as upregulates Akt and cyclic AMP (cAMP) response element binding (CREB) protein which restores function in *N*-methyl-D aspartate (NMDA) receptor and reduces Ca^2+^ influx in schizophrenia, MDD, and AD. CO has a role in NMDA-induced calcium ion (Ca^2+^) influx or excitotoxicity via S-nitrosylation of antiglutamate receptor NMDAR2A (NR2A) subunit and restores synaptic function in AD and SCZ. However, these gas neurotransmitters work on various ways to maintain or restore synaptic plasticity in these neuropsychiatric diseases.

H_2_S is a toxic gas characterized by the smell of rotten eggs. Physiological concentrations of H_2_S selectively increase NMDAR-induced responses as well as the advantageous induction of LTP [7]. One study demonstrated that H_2_S can help maintain amygdala-dependent emotional memory by increasing the function of GluN2B-expressing NMDARs in the amygdala of rats [67]. Pathophysiological concentrations (200 pM) of sodium hydrogen sulfate (NaHS), an H_2_S donor, stimulates seizure-like events in rats *in vivo* and *in vitro*, which may be due to increased neuronal excitation [68]. Previous studies indicated that H_2_S might improve depressive and anxiety-related behaviors in nonstressed rats and mice, but the effects of H_2_S on MDD animal models and the potential mechanism behind these effects are unknown [69]. To understand the actions and underlying mechanisms of H_2_S related to depressive-like behavior, a recent study (intraperitoneally) injected the H_2_S donor NaHS or administered inhaled H_2_S in a chronic unpredictable mild stress (CUMS) model. The role of the mechanistic target of rapamycin (mTOR) signaling pathway and glutamate receptors (Figure 2) in the antidepressant effects of H_2_S was evaluated [70]. The results indicated that a deficiency in endogenous H_2_S in the hippocampus is responsible for the abnormal behaviors associated with CUMS, whereas enhancing hippocampal H_2_S levels by the administration of NaHS or inhalation of H_2_S could correct the depressive-like behaviors of rats within a few hours. This suggests that H_2_S could function as a rapid-onset antidepressant. Additionally, H_2_S could counteract the loss of dendritic spines in the hippocampus that is associated with CUMS [70].

BDNF induces traditional antidepressant actions, and BDNF deletion in the hippocampus weakens antidepressant behavioral responses [71]. Some studies demonstrated that H_2_S reversed the decrease in tropomyosin receptor kinase B (TrKB) receptors induced by CUMS, demonstrating the pivotal role of neurotrophic signaling in the antidepressant effects of H_2_S. These findings are consistent with observations that H_2_S exerted neuroprotective effects against formaldehyde-induced toxicity in PC12 cells via the BDNF–TrKB pathway [72]. This suggests that the synaptogenesis induced by glutamate receptor activation requires the release of BDNF to activate TrKB-mTOR signaling and synaptic protein synthesis (Figure 2). In another study, it was unclear whether the peripheral effects of H_2_S played a role in the antidepressant responses; thus, additional studies are needed [70]. Nevertheless, this study also demonstrated that the acute application of H_2_S, via either the H_2_S donor NaHS or H_2_S gas inhalation, induced robust antidepressant effects that were mediated by activation of the mechanistic target of rapamycin complex 1 (mTORC1) signaling pathway followed by the enhanced synthesis of synaptic proteins containing postsynaptic density protein 95 (PSD95) and synaptophysin. H_2_S also increases the levels of TrKB receptors, which further increases the activity of the GluR1 and GluR2 subunits of AMPARs. An improved understanding of the roles of H_2_S could provide insight into potential therapeutic interventions for depression [70].

Ketamine is a noncompetitive blocker of NMDAR that also stimulates the mTOR signaling pathway and subsequently increases the synthesis of the proteins involved in synapses to induce fast-acting antidepressant effects [73]. A study on ketamine-induced antidepressant effects provided an opportunity to explore the ability of new antidepressants with rapid-acting effects to provide sustained relief and fewer side effects. Various studies have demonstrated a link between H_2_S and the mTOR signaling pathway. A recent study demonstrated that H_2_S could decrease smoking-induced autophagic cell death by activating mTOR [74]. A novel H_2_S-releasing molecule GYY4137 (water-soluble, slow-releasing H_2_S donor) likely protected against high glucose-induced cytotoxicity by activating the mTOR signaling pathway in H9C2 (embryonic cardiomyocyte cell line) cells [75]. Additionally, H_2_S ameliorated hepatic ischemia/reperfusion injury by stimulating phosphorylation of the pyruvate dehydrogenase kinase 1 (PDK-1)/Akt (protein kinase B)/mTOR/70 kDa ribosomal protein S6 kinase (p70S6K) axis [76]. The effects of H_2_S on mTOR activation are consistent with the mechanism of rapid-onset antidepressants [70].

H_2_S is a gasotransmitter that activates the TrKB or mTOR signaling pathways to exert antidepressant effects that are indirectly associated with synaptic protein synthesis or restoration of synaptic plasticity in MDD.

#### 4.1.2. MDD and NO

NO is a highly diffusible and reactive molecule that is synthesized and released with the help of NOSs during the conversion of arginine into citrulline, generating NO in the process [77]. NO mediates the effects of various neurotransmitters, such as norepinephrine, serotonin, glutamate, and dopamine, and thereby plays an essential role in the neurobiology of major depression. Modified NO levels in various brain regions, cerebrospinal fluid (CSF), blood, and exhaled gas have been reported in depression [78]. A meta-analysis revealed disorders in neurooxidative pathways in major depression [79]. Major depression is associated with nitrosative stress, as marked by elevated iNOS function and nitration, as well as by protein nitrosylation [80]. In depression, both neurooxidative and neuronitrative pathways may cause neuroprogression, such as the neuronal dysfunctions caused by oxidative pathways following enhanced neurotoxicity and cytotoxicity, disorders in synaptic plasticity, and reduced neuroprotection [81].

Postmortem studies of patients with MDD revealed reduced neuronal NO synthase levels in the locus coeruleus and a lower number of density of NO synthase-immunoreactive neurons in the hypothalamic nuclei of patients compared with healthy controls [82]. When peripheral NO levels were measured in MDD, some studies reported increased levels [83], whereas other studies found no change [84]. In medication-free depression, various studies found reduced NO levels [85]. In MDD, NO levels were reduced in drug-free patients experiencing depressive episodes in one study [85], but they were either increased or unchanged in another study [84]. Lu et al. demonstrated that NO levels were much higher in MDD patients but then decreased after antidepressant treatment. In the same study, the levels of amino acids, such as citrulline and arginine, were estimated as an index of NO synthesis [78]. In another study, elevated plasma NO levels were reported in male rat models of chronic and unpredictable stress as well as in first-episode MDD patients [86]. The plasma levels of NO metabolites, such as nitrite and nitrate, which reflect plasma NO levels, are also increased in depression [84]. One study described that elevated plasma NO levels in melancholic MDD patients are persistent [78]. This is consistent with previous reports, which also found increased NO plasma levels in MDD patients [87]. Additionally, the elevated NO plasma levels may be associated with suicide attempts in these patients. Modified glutamatergic and decreased GABAergic activity and NO neurotransmission were reported in various brain systems in depression, and this may have a critical effect on the neuronal functions associated with stress responses and mood maintenance [78].

NO is an essential gasotransmitter for neuronal homeostasis, and its upregulation is linked with MDD found in some studies above. Maintaining physiological concentrations of NO could be an effective therapeutic option in MDD.

### 4.2. SCZ

SCZ is a complex psychiatric illness caused by dysregulation of multiple brain neurotransmitter systems, such as those involving dopamine, glutamate, GABA, serotonin, and acetylcholine. Hence, modifications to these neurotransmitter systems [88] have led to hypotheses centering on the expression and functions of neurotransmitter receptors as critical elements of the pathophysiology of this condition; assimilation of signaling mediated by various neurotransmitter receptors is a pivotal step in achieving the functional interactions of receptor activation [89]. Subsequently, modifications of signal integration pathways may have a role to the pathophysiology of SCZ [88]. Gasotransmitters such as H_2_S, NO, and CO have some roles in SCZ.

#### 4.2.1. SCZ and NO

NO is associated with synaptic plasticity, neural plasticity, and cognition [90]. It bolsters the survival and differentiation of neurons as well as exhibits long-lasting events by maintaining transcription factors and altering gene expression. Lower concentrations of NO induce neuroprotective effects and support physiological signaling events, leading to neurotransmission and vasodilatation. In contrast, higher concentrations promote inflammatory effects and are neurotoxic [91]. It was hypothesized that NO could act as a retrograde messenger at synapses, transmitting signals from target neurons back to the synapses and maintaining synaptic plasticity. These same characteristics also allow NO to signal to any local compartment and to cells with defective synaptic activity and NOS expression [92]. Recent evidence revealed roles for NO and related molecules in the pathogenesis of SCZ. Changes in various effects of NO in CNS development may result in neurodevelopmental changes involved in SCZ [92]. NO is associated with many processes in the brain, such as the maintenance of synaptic plasticity, the release of mediators, and the development of nervous tissue [93].

Russian scientists Averbukh et al. (1966) and Bulba et al. (1968) first hypothesized that NO was involved in the onset of SCZ [94]. Studies reporting elevated levels of NO in the postmortem brain tissue [95] and plasma [96] of patients with SCZ also support a link between NOS activity and SCZ. The amount of nNOS differs in patients with SCZ and healthy controls [97]; yet, this issue is conflicted. The nNOS levels in the cortex of the cerebelli of patients with SCZ did not differ from the levels found in those of healthy controls in a study performed by Doyle and Slater in 1995 [98]. In another study, enhanced NO synthase activity was detected in Purkinje cells and the dentate nucleus of patients with SCZ but not with depression [99]. However, data regarding the presence of NOS in the neocortex are inconsistent [100]. Although the upregulated expression of nNOS was reported in the prefrontal cortex in SCZ [100], contrasting data have also been published. Striking data were carried out in the period of investigations of neurons of hypothalamus. The downregulation of nNOS-containing neurons was reported in the periventricular nucleus of patients with SCZ and affective disorders [101]. It was reported that NO in the hypothalamus maintains the synthesis and release of the hormones that maintain the hypothalamic–pituitary–adrenal system (HPAS), including oxytocin, vasopressin, and corticoliberin. The altered production and release of these peptides leads to hyperactivation of HPAS in patients with SCZ [102].

NO levels have also been measured in biological fluids from SCZ patients. The level of NOS and its metabolites in the blood of patients with SCZ and depression has been assessed in many studies, and the results are conflicting [92]. A meta-analysis performed by Maia-de-Oliveira et al. found no significant difference in the NO levels of patients with SCZ and healthy controls. However, higher levels of NO were found in patients treated with antipsychotics, highlighting the influence of these drugs on the metabolism of NOS [103]. Therefore, the enhanced formation of NO does not seem to be caused by NMDARs. This suggests that AMPARs likely play an important role, especially as they are expressed at high levels in patients with SCZ [104]. The subsequent release of NO results in disturbed synaptogenesis and synaptic remodeling as well as in synaptic membrane modification [105].

Antipsychotics modify NO metabolism in the brain. For example, haloperidol suppresses nNOS activity [106]. Hence, the long-term administration of this drug results in nNOS hyperactivity in the striatum of rats [107]. The authors of this study demonstrated that late modification of nNOS activity in the neostriatum during antipsychotic treatment plays an important role in the pathogenesis of late dyskinesia. It is worth repeating that nNOS activity is higher in the plasma of patients with SCZ receiving antipsychotics compared with healthy controls [103]. These studies call into question the influence of nNOS activity in the brains of patients with SCZ [108]. The effects of antipsychotics on other NOS isoforms have also been studied. Clozapine can prevent iNOS activity and reduce microglial inflammation and NO levels in the brain [109]. The effects of antipsychotics on NO metabolism restore the normal function of NMDARs [92].

In summary, SCZ is a neuropsychiatric disorder in which normal synaptic plasticity is hampered. NO plays important roles in maintaining synaptic functions and synaptic plasticity in account of maintaining proper neuronal functioning.

#### 4.2.2. SCZ, H_2_S, and CO

CBS-derived H_2_S is needed for amygdalar synaptic plasticity and fear conditioning in rats. In particular, inhibiting the function of amygdalar CBS prevented activity-stimulated H_2_S production, blocked LTP initiation, and altered cued fear memory in rats [110]. Treatment with an H_2_S donor corrected the LTP and memory impairments caused via CBS inhibition. This CBS inhibition was related to the maintenance of NMDAR function, as the NMDAR-supported synaptic response was lower when CBS was inhibited and the use of a H_2_S donor increased the amplitude of the NMDAR EPSPs (5-enolpyruvylshikimate-3-phosphate) to a level comparable to those of the normal controls. This suggests that H_2_S homeostasis in the brain is critical for the generation of synaptic plasticity and memory. S-Adenosylmethionine (SAM) stimulates CBS activity; it combines with the regulatory C-terminal domain of CBS to activate the generation of endogenous H_2_S. Nevertheless, the mechanisms by which CBS inhibition alters amygdalar synaptic plasticity and memory require further investigation. Activation of NMDAR modulates synaptic plasticity, learning, and memory, and NMDAR hypofunction (Figure 2) was linked to cognitive deficits in aging as well as other psychiatric disorders, such as SCZ [110].

One study showed that prenatal exposure to CO leads to a variety of neurological effects. Lower concentrations of CO lead to a variety of neurobehavioral disorders in rat offspring. Prenatal CO exposure also hampers various neurotransmitters in the growing brains of male rats; low concentrations altered the mesolimbic dopaminergic function and sexual behavior [111]. Changes in cerebellar catecholamines linked these changes to deficits in motor test performance, learning, and memory, as determined by the reduced total GABA content in the cerebelli of 10-day-old rats exposed to CO prenatally. This suggests that GABAergic neurons may have a specific role in CO toxicity. Another study demonstrated that GABAergic neurons may be specifically vulnerable to CO toxicity. Therefore, GABA signaling is modified in neurological disorders, such as SCZ [111]. HO-1 expression in SCZ can be increased by oxidative and inflammatory stimuli [112]. The selective overexpression of HO-1 in the astrocytes of transgenic mice resulted in oxidative stress, lower neuronal reelin content, increased dopamine and serotonin concentrations in the basal ganglia, decreased D1 receptor binding in the nucleus acumens, and altered hippocampal cytoarchitectures. These pathological changes were related to enhanced co-motor activity and reduced proton pump inhibitors but did not affect anxiety or motor balance [113].

H_2_S and CO have a potential role in neuronal homeostasis, and maintaining proper amounts of these gasotransmitters is a crucial factor in case of SCZ.

### 4.3. BD

BD is a severe neuropsychiatric condition that results in repeated episodes of mania, which are pathologically energized states characterized by poor judgment, euphoria, irritability, and in depressive episodes, which are characterized by dispiriting moods, decreased energy, volitional states, and decreased cognitive capacity [114]. The gasotransmitter NO is related to the BD which is briefly discussed below.

#### 4.3.1. BD and NO

Modified NO signaling, which directly affects neurotransmitter release and synaptic plasticity cascades, has been demonstrated in BD. Lithium maintains NO levels in preclinical models. However, the effects of lithium ion NO levels have not been studied in humans [115]. Upregulated NO levels were reported during various mood states [116], particularly depressive episodes [117]. The NO pathway is particularly important in neuropsychiatric disorders. Altered NO levels affect neurotransmitter release [90] and synaptic plasticity [118]. High concentrations of NO have dose-dependent neurotoxic effects, whereas physiological concentrations play neuromodulatory and neuroprotective roles [91]. The neuroprotective effects of NO include reducing Ca^2+^ influx and subsequent cell death (Figure 2) [119]. Additionally, NO upregulates the expression of the neuroprotective proteins Akt and CREB (Figure 2) [120] and the potent antioxidant bilirubin [121].

NO effects are persistent with neuroprotective as well as neurotrophic roles of lithium [122]. Lithium is the standard and first-line treatment option for BD [123]. Its mechanism of action is complex and involves multiple intracellular signaling pathways. Various animal studies have revealed that lithium maintains central and peripheral NO levels [124] and significantly increases NO levels in BD depression after 6 weeks of treatment. However, there was no marked difference in NO levels between unmedicated BD patients and matched healthy controls [122]. These experiments suggest that NO levels may be maintained by lithium in humans. Along these lines, an increasing body of preclinical evidence suggests that lithium can directly target NO signaling [124]. For example, lithium upregulates *NOS* messenger RNA (mRNA) expression in glial cultured cells [125], the hypothalamus [126], and the hippocampus [127] and also increases cortical NO metabolites in rodents. Other preclinical studies showed that lithium reduces NO metabolites [128] in rat neural tissues [129]. The upregulated NO levels were not associated with clinical improvement, increasing the possibility that the effects of lithium ion NO may be an epiphenomenon or an intermediate pathway for the antidepressant effect [115].

In a recent study, the plasma NO levels in BD patients did not differ from those of healthy controls [115]. In terms of mood disorders, studies on NO have yielded mixed results. At the same time, several studies reported enhanced NO levels in BD [116], and another study that analyzed NO levels during a depressive episode in BD [117] showed elevated NO metabolite levels in subjects with BD receiving multiple medications that can influence NO [103]. The fact that the sample contained drug-free patients with a short duration of illness means that it is possible that NO plays a more important role in BD late in the course of the illness, after patients have been exposed to chronic insults such as repetitive episodes, medications, and comorbidities. Additionally, the number of previous mood episodes was positively correlated with NO levels in BD [130]. These studies may support a crucial role for NO signaling in the trophic and neuroprotective effects of lithium in BD and other neuropsychiatric disorders [115].

In conclusion, the gasotransmitter NO plays a pivotal role in maintaining neural plasticity and proper neuronal functioning in the service of preserving the activity of the CNS in BD.

### 4.4. AD

AD is characterized by the loss of neurons and synapses in the hippocampus, cerebral cortex, and subcortical regions, as well as the formation of amyloid beta (A*β*) plaques and neurofibrillary lesions. The main protein component of plaques is amyloid-*β*, which is derived from the proteolytic cleavage of amyloid precursor protein (APP). Mutations associated with early-onset of familial AD increase A*β* production. A*β* isolated directly from human AD brains caused impaired synaptic plasticity and memory in rodents [131]. Synaptic activity and chronic sleep restriction increase the amount of A*β* in brain and intestinal fluid, as well as plaque formation in APP transgenic mice [132]. AD is related with some gasotransmitters such as CO and NO, which is discussed briefly in the following subsections.

#### 4.4.1. AD and CO

As discussed above, mammalian tissues express two isoforms of heme oxygenase: HO-1 and HO-2. The third isoform, HO-3, is a retrotransposition of the *HO-2* gene and is only found in rats [133]. The basal expression of HO-1 in the normal brain is restricted to small groups of scattered neurons and neuroglia [134], whereas HO-2 is more broadly expressed across the neuraxis [60]. HO-1 is a 32 kDa protein that catalyzes the breakdown of heme to free iron, CO, and biliverdin. In “stressed” astroglia, HO-1 hyperactivity stimulates mitochondrial iron sequestration and macroautophagy, which may be responsible for the pathological iron accumulation and bioenergetic failure observed in AD as well as in other neurodevelopmental conditions. The expression of glial HO-1 may also affect neuroplasticity and cell survival by modulating the brain sterol metabolism and the proteasomal deterioration of neurotoxic proteins [135].

HO-1 immunoreactivity in glia increases progressively as aging progresses in the normal human brain [136]. HO-1 deteriorates as neural tissue senesces, which may be responsible for the biogenesis of the corpora amylacea and glycoprotein-rich inclusions generally encountered in aging mammalian cells [137]. The number of glial fibrillary acid protein- (GFAP-) positive astrocytes that express HO-1 is increased significantly in the hippocampus and cerebral cortex of patients with AD compared with age-matched, nondemented controls. An excessive increase in glial HO-1 levels is already apparent in the brains of subjects with mild cognitive impairment (MCI), which is a common precursor or indication of incipient AD [138]. In the temporal cortex of patients with MCI, the number of astrocytes with immunoreactivity for HO-1 is related to the degree of neurofibrillary pathology and also the reductions in scores on tests of global cognition, episodic memory, semantic memory, and working memory. Similarly, HO-1 expression in astroglia is associated with lower scores for global cognition, perceptual speed, and semantic memory.

Elevated CO is found in the above studies in AD, and regulating physiological levels of CO could be a therapeutic option in case of AD.

#### 4.4.2. AD and NO

Deficits in synaptic plasticity are increasingly recognized as causes of memory loss in AD [139]. However, the early mechanisms driving synaptic pathophysiology are poorly understood. Short-term plasticity and long-term plasticity are calcium-dependent processes that can be altered by second messengers, such as NO. NO is produced by NOS via NMDAR-mediated calcium entry [140]. NO signaling is involved in neurodegenerative diseases via the formation of reactive nitrogen species and cGMP signaling cascades [141]. NO also has neuroprotective effects, as shown in AD mouse models in which it reduces cell loss and tau pathology [142]. In AD models, NO can be altered via various mechanisms. For example, the NMDAR-mediated calcium entry that activates NOS is augmented by abnormal ryanodine receptor- (RyR-) mediated calcium-induced calcium release [143]. NOS protein levels and RyR levels are also increased in both AD mouse models and human AD brains [144]. In presynaptic AD mice, these conditions that amplify NO levels occur alongside exaggerated hippocampal synaptic depression. These deficits occur when homeostasis is challenged, such as in the presence of reduced RyR-calcium release. Although the hippocampal network and cognitive performance appear normal, they clearly are not [145].

In one study, the primary site of NO regulation in 3×-Tg-AD mice was the presynaptic terminals, where it increased evoked and spontaneous vesicle release, as determined by PPF assays and spontaneous vesicle-release properties [146]. In this study, NO could increase transmitter release via cGMP signaling as well as the S-nitrosylation of synaptic proteins, which increases the presynaptic binding of syntaxin to VAMP and SNAPZS [147]. NO also alters the magnitude of vesicular release by converting reserve pool vesicles into easily releasable vesicles [148]. NO can also increase RyR channel opening, possibly via S-nitrosylation. The opposing interactions between enhanced RyR-calcium signaling and increased nNOS expression in AD neurons can sustain enhanced NO production or synthesis and also increase presynaptic gain. Postsynaptically, the neuroprotective characteristics of NO curb the excessive NMDAR-induced calcium influx and excitotoxicity by causing S-nitrosylation of the antiglutamate receptor NMDAR2A (NR2A) subunit (Figure 2). At the same time, the S-nitrosylation of caspase-3, -8, and -9 decreases apoptosis. The enhanced nNOS levels and NO activity in AD brains may have a neuroprotective role, as demonstrated by the selectively spared NOS-positive neurons in AD. Hence, constant increases in NO have harmful effects, such as oxidative stress, the fragmentation loss of synaptic functioning, and apoptosis [141].

In summary, AD is a major neuropsychiatric disorder, and the regulation of NO is essential for maintaining proper neuronal functioning and synaptic plasticity in the CNS during AD.

## 5. Future Directions

Gasotransmitters are the essential molecules that help regulate synaptic and neuroplasticity in the CNS. Three gas neurotransmitters, H_2_S, NO, and CO, were discussed briefly. These gasotransmitters are related to neuropsychiatric conditions. For example, H_2_S downregulation was involved with the progression of MDD in one study, but the molecular mechanisms by which reduced H_2_S levels lead to MDD need to be studied further [69, 70]. In terms of other gasotransmitters, higher NO levels were reported in the postmortem brains of SCZ patients [103]; thus, downregulation or modified regulation could be a therapeutic option for treating SCZ. NO levels are also altered in BD patients, and this could facilitate the identification of therapies to treat these neuropsychiatric conditions. Additionally, increased levels of HO-1 or CO were observed in AD patients, which highlights how gasotransmitters may be involved with neuropsychiatric conditions and how regulating these gasotransmitters could help treat these disorders.

## 6. Conclusion

Maintaining synaptic plasticity is crucial for regulating neuronal health and homeostasis. Neuronal functioning declines over time or under stressful conditions whereas many reactive species can lead to different neuropsychiatric conditions, such as SCZ, MDD, BD, and AD. Several gasotransmitters, such as H_2_S, NO, and CO, balance synaptic plasticity when the normal condition is altered directly or indirectly. However, as the mechanisms or pathways through which they act are poorly understood, further studies are needed. Because the up- or downregulation of these gasotransmitters is responsible for causing the pathological conditions that lead to neuropsychiatric diseases, the normalization of their levels could exert protective effects. Hence, ensuring that the levels of these gasotransmitters are appropriate could help in the treatment of neuropsychiatric conditions. Moreover, improvements in our understanding of these pathways may lead to the identification of new therapeutic options for these neuropsychiatric conditions.

## Abbreviations

AD: Alzheimer's disease
AMPA: *α*-Amino-3-hydroxy-5-methyl-isoxazole-4-propanoic acid
AMPARs: *α*-Amino-3-hydroxy-5-methyl-isoxazole-4-propanoic acid receptors
A*β*: Amyloid beta
APP: Amyloid precursor protein
BD: Bipolar disorder
BDNF: Brain derivative neurotrophic factor
CA3: Cornu Ammonis 3
CaMKII: Calcium/calmodulin-dependent kinase II
CREB: Cyclic AMP response element binding protein
CO: Carbon monoxide
Ca^2+^: Calcium ion
CBS: Cystathionine *β*-synthase
CSE: Cystathionine *γ*-lyase
CAT: Cysteine aminotransferase
cGMP: Cyclic guanosine monophosphate
cAMP: Cyclic adenosine monophosphate
CUMS: Chronic unpredictable mild stress
DAAO: D-Amino acid oxidase
DRG: Dorsal root ganglion
ERK: Extracellular signal-regulated kinase
EPAC: Exchange protein stimulated by cAMP
GABA: Gamma amino butyric acid
GFAP: Glial fibrillary acid protein
GSH: Glutathione
GSSH: Glutathione persulfide
H_2_S_n_: Polysulfide
H_2_S: Hydrogen sulfide
HO-1: Hemeoxygenase-1
HO-2: Hemeoxygenase-2
HO-3: Hemeoxygenase-3
HPAS: Hypothalamic–pituitary–adrenal system
JNK: C-Jun *N*-terminal kinase
LTP: Long-term potentiation
LTD: Long-term depression
MDD: Major depressive disorder
MAPK: Mitogen-activated protein kinases
3-MST: 3-Mercaptopyruvate sulfurtransferase
mTOR: Mechanistic target of rapamycin
mRNA: Messenger ribonucleic acid
MCI: Mild cognitive impairment
NMDA: *N*-Methyl-D-aspartate
NMDARs: *N*-Methyl-D-aspartate receptors
NO: Nitric oxide
NOS: Nitric oxide synthase
NaHS: Sodium hydrogen sulfate
NSCs: Neural stem cells
Nrf2: Nuclear factor erythroid 2-related factor 2
PKA: Protein kinase A
RyR: Ryanodine receptor
SCZ: Schizophrenia
SSRIs: Serotonin reuptake inhibitors
SAM: S-Adenosylmethionine
siRNA: Small interfering RNA
TrkB: Tropomyosin receptor kinase B
TRPA1: Transient receptor potential ankyrin 1
VGCC: Voltage-gated calcium channels
VEGF: Vascular endothelial growth factor
WHO: World Health Organization.
